# Adoptive parenting and attachment: association of the internal working models between adoptive mothers and their late-adopted children during adolescence

**DOI:** 10.3389/fpsyg.2015.01433

**Published:** 2015-09-23

**Authors:** Cecilia S. Pace, Simona Di Folco, Viviana Guerriero, Alessandra Santona, Grazia Terrone

**Affiliations:** ^1^Department of Educational Sciences, University of GenoaGenoa, Italy; ^2^Department of Pedagogy, Psychology, Philosophy, University of CagliariCagliari, Italy; ^3^Department of Dynamic and Clinical Psychology, Sapienza University of RomeRome, Italy; ^4^Department of Psychology, University of Milano-Bicocca, MilanoItaly; ^5^Department of Humanities, Literature, Cultural Heritage, University of FoggiaFoggia, Italy

**Keywords:** attachment, adoption, adolescence, internal working models (IWMs), Friend and Family Interview

## Abstract

**Introduction:** Recent literature has shown that the good outcome of adoption would mostly depend on the quality of adoptive parenting, which is strongly associated with the security of parental internal working models (IWMs) of attachment. Specifically, attachment states-of-mind of adoptive mothers classified as free and autonomous and without lack of resolution of loss or trauma could represent a good protective factor for adopted children, previously maltreated and neglected. While most research on adoptive families focused on pre-school and school-aged children, the aim of this study was to assess the concordance of IWMs of attachment in adoptive dyads during adolescence.

**Method:** Our pilot-study involved 76 participants: 30 adoptive mothers (mean age = 51.5 ± 4.3), and their 46 late-adopted adolescents (mean age = 13.9 ± 1.6), who were all aged 4–9 years old at time of adoption (mean age = 6.3 ± 1.5). Attachment representations of adopted adolescents were assessed by the Friend and Family Interview (FFI), while adoptive mothers’ state-of-mind with respect to attachment was classified by the Adult Attachment Interview (AAI). Adolescents’ verbal intelligence was controlled for.

**Results:** Late-adopted adolescents were classified as follows: 67% secure, 26% dismissing, and 7% preoccupied in the FFI, while their adoptive mothers’ AAI classifications were 70% free-autonomous, 7% dismissing, and 23% unresolved. We found a significant concordance of 70% (32 dyads) between the secure–insecure FFI and AAI classifications. Specifically adoptive mothers with high coherence of transcript and low unresolved loss tend to have late-adopted children with high secure attachment, even if the adolescents’ verbal intelligence made a significant contribution to this prediction.

**Discussion:** Our results provides an empirical contribution to the literature concerning the concordance of attachment in adoptive dyads, highlighting the beneficial impact of highly coherent states-of-mind of adoptive mothers on the attachment representations of their late-adopted adolescent children.

## Introduction

Attachment theory stressed the importance of early parent–child relationships for normative development of socio-emotional functioning across the life span (Thompson, 1999). These relationships play a significant role on the development of a child’s internal working models (IWMs) of the self, others, and relationships influencing the child’s attachment security (Bowlby, 1969), and they guide the construction and the expectations of future social interactions. IWMs of caregivers are expected to affect parenting and caregiving transactions that mothers enact both consciously and unconsciously in their interactions with the child (Bretherton and Munholland, 2008; Dazzi and Zavattini, 2011; Velotti et al., 2011). Literature also established that parents’ IWMs, manifested in discussions about childhood experiences during the Adult Attachment Interview (AAI; George et al., unpublished), predicted the quality of the infant-parent attachment relationship as observed in Ainsworth’s Strange Situation procedure (SSP; Ainsworth et al., 1978; van IJzendoorn, 1995; Steele et al., 1996; Tini et al., 2003; Verhage et al., 2015).

Overall, literature suggested parents’ IWMs that form the basis of parenting behaviors (sensitivity, attunement, monitoring, etc.) may influence the child’s IWMs from early childhood to adolescence (Karavasilis et al., 2003; Gamble and Roberts, 2005; Bosmans et al., 2006; Gallarin and Alonso-Arbiol, 2012). However, only a few studies assessed the concordance of attachment representations between parent–children dyads (mostly “mother–son”) in this stage of life (Rosenstein and Horowitz, 1996; Zimmerman et al., 1997; Allen et al., 2003; Scharf et al., 2012), providing evidence of a weak to moderate intergenerational effect. Further, the measurement of attachment in adolescence presents some weaknesses. Literature showed that, when assessing IWMs, the AAI (Bakermans-Kranenburg and van IJzendoorn, 2009) and the Attachment Interview for Childhood and Adolescence (AICA; Ammaniti et al., unpublished) were used, but without taking into account the specificity of this stage. Moreover, conscious attachment styles may be captured by questionnaires, such as the Inventory of Parent and Peer Attachment (IPPA; Armsden and Greenberg, 1987; Pace et al., 2011), which measures attachment security and its factors (trust, communication, and alienation) in adolescence, but presents obvious limitations characterizing self-reports. Finally, in recent years, the Friends and Family Interview (FFI; Steele and Steele, 2005) were developed to assess attachment representations in late childhood and adolescence, including important relationships beyond the child–parent relationships and provided encouraging results (Kriss et al., 2012; Pace, 2014; Steele et al., unpublished).

Growing literature has recently examined attachment among adoptive families in the years following adoption. Adoption in Italy is a very common phenomenon. In the period between 2000 and 2013, 42,048 children were legally authorized to enter the country for adoption. Children were adopted by 33,820 couples with an average of 1.24 children per couple (Italian Commission for International Adoptions, 2013). Internationally adopted children’s mean age at arrival was 5.5 years with four children out of 10 (42.1%) between 1 and 4 years old and 43.8% of adopted children between 5 and 9 years old; 8.8% were 10 years or older, while only 5.4% of adopted children were younger than 1 year old.

As reported in a 2009 meta-analysis (van den Dries et al., 2009), children who were adopted before 12 months of age were as securely attached as their non-adopted peers, whereas children adopted after their first birthday were less securely attached than non-adopted children (*d* = 0.80, CI = 0.49–1.12). Moreover, adopted children showed more disorganized attachment compared to their non-adopted peers (*d* = 0.36, CI = 0.04–0.68), but they were less often disorganized compared to institutionalized children. Thus, early adoption is a considerable and effective intervention in the domain of attachment relationships (Lionetti, 2014).

However, as mentioned above, in the Italian adoption practice, almost 95% of internationally adopted children were placed after 1 year of life, and, thus, they should be considered as late-adopted children (Howe, 1998). Late adoption represents an exceptional intervention aimed at influencing and reprocessing representations of children who often suffered traumas, abuse, and neglect in their early infancy or childhood (Rutter and O’Connor, 2004; Juffer and van IJzendoorn, 2005; van IJzendoorn and Juffer, 2006; Dozier and Rutter, 2008). Furthermore, no differences were found in the attachment patterns between international and domestic adopted children probably because similar early negative experiences were suffered by the adopted children, independently from the type of adoption (van den Dries et al., 2009). On the one hand, late-adoption represents a window for the investigation of the impact of children’s negative pre-adoption experiences on the development of insecure-disorganized IWMs of attachment (Steele et al., 2007; Pace et al., 2015b). On the other hand, late adoption embodies the opportunity for children’s schemas to be revised and reprocessed based on the “new” relationships with adoptive caregivers (Steele et al., 2003, 2008; Juffer and van IJzendoorn, 2005). Some studies highlighted that previously maltreated and neglected children placed after 4 years of age and assessed both through a behavioral procedure and narrative tasks (Pace and Zavattini, 2011; Pace et al., 2012) showed increasing attachment security over 2 years after adoption. Additional findings showed that late adopted children improve markedly in the positive representations of the self, the caregiver, and in the relationship with others and also in the narrative’s coherence (Hodges et al., 2003; Kaniuk et al., 2004). As suggested from these empirical findings, further positive revision may be possible, even in older adopted children, and, therefore, exploring which parental characteristics could foster their “earned” security deserves attention (Pace et al., 2012). In the Attachment Representations and Adoption Outcome study (Steele et al., 2003, 2007) mothers’ insecurity of attachment (either dismissing or preoccupied) as assessed by the AAI 3 months after adoption was correlated with children’s (4–8 years old) negative narratives and disorganized or bizarre themes proposed in an attachment story completion. In addition, children with unresolved adoptive mothers failed to establish secure attachment and positive representations of self and others (Steele et al., 2003). Both parents’ attachment insecurity was strongly associated with high levels of disorganization or insecurity in the adoptees and confirmed even 2 years later. When neither parents’ AAI was secure at the time of placement, 2 years later 86% of adopted children scored high for disorganization (Steele et al., 2008). Veríssimo and Salvaterra (2006), assessing a sample of Portuguese children adopted between 3 weeks and 47 months of age, found that the scores reflecting the presence and quality of maternal secure representations predicted the level of attachment security of adopted children, as measured by Secure Base Scripts (SBS; Waters and Waters, 2006) and assessed by the Attachment Behavior Q-Set (AQS; Waters, 1995) (Spearman rho = 0.38, *p* < 0.01) with no correlations either with child’s age at time of adoption or the child’s age at time of assessment. Barone and Lionetti (2012), assessing parents’ attachment state-of-mind using the AAI and children’s (3–5 years old) attachment patterns, administered a doll story completion task 12–18 months after placement and found 80% concordance with respect to two attachment classifications in mother–child dyads and 60% concordance with respect to three-way attachment classification. Concerning father–child dyads, no significant associations were found. Pace and Zavattini (2011) and Pace et al. (2012) found that late-adopted children (4–7 years old) who presented significant enhancing attachment security were predominantly placed with secure adoptive mothers (*p* < 0.05). However, the concordance between the adoptive mothers’ attachment representations and their adopted children’s narratives on the two-way system (secure vs. insecure) was not significant (56%).

All these studies focus on the few years after late-adopted children’s placement, usually during middle childhood, while only a few studies examine what happens during later stages, such as late-childhood and adolescence. These studies show a percentage of secure attachment of adolescent adoptees that range between 32% (Beijersbergen et al., 2012) and 63% (Riva Crugnola et al., 2009), using the AAI or AICA and from 32% (Escobar and Santelices, 2013) to 51% (Groza et al., 2012) and 60% (Barcons et al., 2012), using the FFI. Most of these studies found no unresolved or disorganized (U/D) classifications either by the AAI or the FFI (only 2% in Barcons et al., 2012), meaning that adoptees were able to develop an organized attachment strategy, despite their early negative experiences. This data is worthwhile given that the disorganized attachment could be considered a strong predictor of short- and long-term psychopathological problems (van IJzendoorn et al., 1999; West et al., 2001). Except for Escobar and Santelices (2013), no study found a significant association between the age of adoption and attachment patterns, meaning that older age at adoption did not automatically imply high attachment insecurity.

Given that parenting seems to continue to influence children’s attachment representations, even during adolescence (Hoeve et al., 2011), attachment researchers have recently questioned the role of adoptive parents in influencing attachment in adopted adolescents. A longitudinal adoption study (Beijersbergen et al., 2012), assessed through the AAI, revealed that mothers of secure adolescents showed significantly more sensitive support during conflicts than did mothers of insecure adolescents. The authors concluded that both early and later maternal sensitive support were important for continuity of secure attachment for the first 14 years of life of early adopted adolescents. Another study (Riva Crugnola et al., 2009) assessing attachment in adopted adolescents and their adoptive parents, using the AAI and the AICA (Ammaniti et al., unpublished), did not find any significant concordance between mother–child and father–child two-way attachment systems. However, they suggested that the majority of parents who were secure with respect to attachment had children who were also secure, while those who were insecure had adopted children who were equally distributed in the two-way attachment classifications (secure vs. insecure). Limitations of this study, however, should be addressed due to both the wide variability of the sample characteristics and the lack of control for background variables.

Given the growing importance of assessing attachment bonds between adoptive parents and their children, especially in adolescence where there is a shortage of literature, in the current correlational study we investigated attachment concordance between late-adopted adolescents and their adoptive mothers. We expected correspondence between mothers’ AAI attachment representations and children’s FFI attachment representations (AAI and FFI categories and state-of-mind scales), mostly at the level of secure vs. insecure partition, as we controlled for demographic variables, adolescents’ verbal cognitive status, that can foster secure attachment patterns (West et al., 2013), and maternal psychopathological symptoms.

## Materials and Methods

### Participants

The adoptive families were recruited through two authorized international adoption agencies [e.g., Centro Italiano di Aiuti all’Infanzia-CIAI (Italian Center for Supporting Childhood) and Associazione Teresa Scalfati (Teresa Scalfati’s Association)], an association supporting adoptive families [Genitori si Diventa (Becoming Parents)] and the social-health service specialized on adoption working in Rome. All the participants lived in the following cities of the Center of Italy: Rome, L’Aquila, and Teramo.

The eligible criteria for this study were the following: age range of late-adopted adolescents between 11 and 16 years old, age of adoption after 4 years of age, length of placement equal to 4 years at least (considered a sufficient length of time for stabilizing adoptive child–parent relationships, van den Dries et al., 2009), absence of children’s special needs, absence of maternal clinical diseases, parents with medium-to-high education level, married couples still living together, and families living in urban contexts.

This study included 76 participants: 46 late-adopted adolescents (23 female) and their 30 adoptive mothers. Of the adolescents, 14 were “only” children, while 32 were siblings both involved in the study. 21 mother–child dyads had already participated in a longitudinal study (Pace and Zavattini, 2011; Pace et al., 2012). The pre-adoption histories of the adoptees were characterized by severe adversities, such as serious neglect, physical maltreatment, sexual abuse, and widely variable periods of institutionalization.

### Variables and Measures

#### Late-adopted Adolescents

##### Adolescents’ attachment representations

Attachment representations of adolescents were assessed using the Italian version of the FFI (Steele and Steele, 2005), authorized by the author Howard Steele. The FFI is a semi-structured interview informed by, yet distinct from, the AAI (Main et al., 2008). Interviews are video-recorded and transcribed verbatim.

The FFI’s coding system comprises eight scales, each including subscales as follows: (1) *Coherence*, based on Grice’s well-known maxims of good conversation—truth, economy, relation, and manner, and overall coherence; (2) *Reflective Functioning*—developmental perspective, theory of mind, diversity of feelings; (3) *Evidence of Secure Base*—father, mother, and other significant figure; (4) *Evidence of Self–Esteem*—social competence, school competence, and self-regard; (5) *Peer Relations*—frequency of contact and quality of best friendship; (6) *Sibling Relations*—warmth, hostility and rivalry; (7) *Anxieties and Defense*—idealization, role reversal, anger, derogation, adaptive response; and (8) *Differentiation of Parental Representations*.

The interview also includes the following global *attachment classifications* (Steele et al., unpublished): (1) *secure* classification indicates that the person’s narrative reflects flexibility, ease, and ability to turn to others for support when in distress; (2) *insecure-dismissing* classification describes people who use derogation or idealization as a defense and show restriction when they have to acknowledge or express distressing feelings; (3) *insecure-preoccupied* classification describes adolescents rated highly in anger or passivity; and finally, (4) *insecure-disorganized* classification describes people showing some lapses in monitoring or reasoning as well as contradictory or incompatible strategies in the attachment narratives.

The scales are scored on a 7-point scale from 1 to 4 (1 = no evidence; 2 = mild evidence; 3 = moderate evidence; 4 = marked evidence), including mid-points.

In the FFI coding system, the interviews have both a final classification (the above-mentioned secure, dismissing, preoccupied, and disorganized categories) like in the AAI and a scoring (1–4) for each classification, which is unlike the AAI. This double coding system captures attachment representations both at categorical and dimensional levels.

For this study, two blinded raters (both trained by Howard Steele and reliable coders for the FFI) coded 14 of the 46 interviews (30.4%). Inter-rater agreement was 100% (*k* = 1, *p* < 0.001) on the four-way classification system (secure, dismissing, preoccupied, and disorganized). Spearman’s rho correlations for the five coherence scales ranged from 0.66 for the relation scale (*p* < 0.05) to 0.86 for the manner one (*p* < 0.01). The other FFIs were coded only by one trained coder. To our knowledge, this is the first study assessing attachment representations of late-adopted adolescents with the FFI in Italy.

##### Adolescents’ Cognitive Status

Given the contrasting findings on the links between attachment representations and cognitive level of participants at developmental stages (Steele and Steele, 2005; Stievenart et al., 2011; Beijersbergen et al., 2012; West et al., 2013), we assessed the verbal intelligence of late-adopted adolescents.

Participants’ verbal IQ was measured by the vocabulary subtest from the Wechsler Intelligence Scale for Children (verbal WISC-III, Wechsler, 1991; Italian validation, Orsini and Picone, 2006) for participants aged between 6 and 16 years and 11 months. The verbal WISC III consists of the following subtests: information, similarities, arithmetic reasoning, vocabulary, comprehension (CV), memory figures. The child’s verbal IQ is obtained from the sum of the weighted points of the first five verbal subtests, while the factor score of verbal CV is obtained based on the weighted score received in the last subtest.

#### Adoptive Mothers

##### Maternal attachment states of mind

The states of mind with respect to attachment of adoptive mothers were assessed through the AAI (George et al., unpublished; Main et al., unpublished), a well-known and semi-structured interview with 20 questions lasting approximately 1 h. The AAIs are audio-recorded and transcribed verbatim.

The transcripts were used to assess possible past experiences with attachment figures in infancy (Loving, Rejection, Neglecting, Role Reversal, and Pressure to Achieve) and current states of mind (Idealization, Lack of Memory, Anger, Derogation, Passivity, Transcript Coherence, Mental Coherence, Metacognitive Monitoring, Fear of Loss, Unresolved Loss, Unresolved Trauma) on 25 1-to-9 scales.

The coding system classifies attachment states of mind into one of three principal categories: (1) *free-autonomous and secure* (F/A), where individuals freely describe their attachment experiences with balance and coherence; (2) *insecure-dismissing* (Ds), where they are unable to give evidence for the positive evaluations of their parents showing idealization or normalization strategies; (3) *insecure-preoccupied* (P), where they use angry or vague language when talking about their attachment relationships. One of two transversal categories can also be added: *insecure-unresolved/disorganized loss or trauma* (U), where transcripts presented lapses in monitoring of reasoning or discourse or reports of extreme behavioral reactions during discussion of these specific topics, or *cannot classify* (CC), where completely contradictory attachment patterns (e.g., dismissing/entangled) emerged.

Psychometric studies in many countries have shown that attachment classifications provided by the AAI are steady across periods of up to 15 months and are independent of the interviewer. The AAI categories were not correlated with the interviewees’ cognitive level, social desirability, memory, or general discourse style (Bakermans-Kranenburg and van IJzendoorn, 1993; Crowell et al., 1996).

For our study, all the AAIs were coded by a reliable coder. For inter-rater reliability, 10 interviews (30%) were also classified by another expert evaluator. Both coders were trained by Deborah Jacobvitz and Nino Dazzi and they were provided with AAI’s reliability and unaware of the other data collected. Inter-rater agreement was 88% (*k* = 0.77, *p* < 0.01) for four-way classifications (free-autonomous, dismissing, entangled, or unresolved). Spearman’s rho correlation of 0.72 was found for the coherence of transcript’s scale (*p* < 0.05), 0.74 for coherence of mind (*p* < 0.05), and 0.97 for unresolved loss (*p* < 0.01).

##### Socio-demographic and Adoption Data

Ad-hoc questions were developed for this research and they were answered by adoptive mothers to collect personal data (age of birth, education level, year of marriage, etc.) and information concerning the details of adoption (children’s age at arrival, country of origin, length of adoption, pre-adoption information, etc.). A part of this sheet was designed to investigate the children pre-adoption histories, especially the motivations for which they were placed for adoption (e.g., parental abandonment, death, drug abuse, etc.) and the events leading to change of guardianship, such as neglect, physical and sexual abuse, institutionalization, and multiple placements. In the last part we asked about physical condition, mental retardation, and psychiatric diagnoses of the children.

##### Maternal psychopathology

Before adoption, parents seeking to adopt were assessed to examine their psychopathological risk, but at the time of this study’s assessment, some years had passed since this pre-adoption selection. Therefore we checked the psychopathology level of adoptive mothers to ensure they were free from mental disorder symptoms and no psychological problems had emerged after adoption.

Mental health problems of mothers were measured using the Symptom Checklist 90 (SCL-90-R; Derogatis, 1994), a 90-item standardized instrument designed to measure current symptom severity grouped in 10 main symptom dimensions (somatization, obsession-compulsion, interpersonal sensitivity, depression, anxiety, hostility, phobic anxiety, paranoid ideation, psychoticism, and other symptoms, such as problems with sleep) and an index of psychopathology (Global Severity Index, GSI). This measure provides a reliable estimate of the likelihood of being diagnosed with a mental health disorder (*T* score above 63 on the GSI for any two symptom dimensions).

### Procedure

The data were collected during a session lasting approximately 2 h at the university’s laboratory. Mothers and children were assessed separately: the FFI and the WISC-III were administered to the adolescents, while the AAI, the socio-demographic questionnaire, adoption sheet, and the SCL-90 were used with their adoptive mothers. Participation in this research was voluntary. Before the session, written informed consent was obtained from all families. The research protocol had been previously approved by the University Ethical Committee.

### Data Analysis

Results were analyzed using the Statistical Package for Social Science (SPSS, Version 21.0). We decided to use primarily non-parametric tests, which are appropriate for variables of this type because they do not require that the sample is drawn from a normally distributed population (Siegel and Castellan, 1988). The significance level for all analyses was set at *p* < 0.05.

First, we presented descriptive statistics of all of the study variables and then we investigated whether FFI and AAI classifications were associated with background and control variables. Next, we tested the study’s hypotheses with respect to the association of attachment between mothers and late-adopted adolescents. Specifically:

(1) We examined the concordance between adoptive mothers’ AAI categories and late-adopted adolescents’ FFI categories on the two-way system (secure–insecure) and, when possible, three-way (secure, dismissing, and preoccupied) and four-way systems (secure, dismissing, preoccupied, and disorganized). Using multinomial logistic regression analyses, we then tested whether FFI attachment category of late-adopted adolescents could be predicted by taking into account mothers’ AAI categories.
(2) We examined the correlation between late-adopted adolescents’ FFI scales and adoptive mothers’ AAI state-of-mind scales (especially coherence scales considered to be the best predictors of free-autonomous classifications). Using linear regression analyses, we then tested whether FFI scales of late-adopted adolescents could be predicted by taking into account mothers’ AAI state-of-mind scales.

## Results

### Descriptive Variables

**Table 1** presents descriptive results concerning background variables and attachment classification for both adolescents and mothers.

**Table 1:** Child and mothers demographic and adoption characteristics.

	Range	Mean	*SD*
**Late-adopted adolescents (*n* = 46)**			
Children’s age at adoption (years)	4–9	6.30	1.52
Children’s age at assessment (years)	11.11–16.11	13.9	1.58
Length of adoptive placement (years)	4–11	7.6	1.46
Lenght of istituzionalization (months)	6–72	31.56	19.67
Verbal IQ	77–154	96.33	25.18
		***N***	***%***
Children’s sex	Boys	23	50
	Girls	23	50
Type of adoption	Domestic	4	8.7
	International	42	91.3
Children’s continent of origin	South America	21	50
	Eastern Europe	10	28.8
	Africa	6	14.3
	Asia	5	11.9
Level of schooling	Middle school	39	84.8
	High school	7	15.2
Friend and Family Interview (FFI) classifications	*S*	31	67.4
	*Ds*	12	26.1
	*P*	3	6.5
	*D*	0	0
**Adoptive mothers (*n* = 30)**			
Mothers’ age	44–59	51.47	4.26
Length of marriage	12–31	20.46	5.43
Years of education	13–21	15.87	2.82
SCL-90 (GSI)	4–62	50.47	5.17
Level of education		***N***	***%***
	High school	14	46.7
	College/Post-graduate	16	53.3
Adult Attachment Interview (AAI) classifications	*F/A*	21	70
	*Ds*	2	6.7
	*P*	0	0
	*U/D*	7	23.3

#### Late Adopted Adolescents

The statistical analyses with adolescents’ FFI categories were conducted using a secure vs. insecure partition with preoccupied adolescents included in the insecure group together with dismissing ones. Adolescents’ FFI classifications were not associated with gender (*p* = 0.35), continent of origin (*p* = 0.10), age at arrival (*p* = 0.50), length of adoption (*p* = 0.60), age at assessment (*p* = 0.27), educational level (*p* = 0.26), or presence or absence of siblings (*p* = 0.69). However, secure adolescents (*M* = 104.39, *SD* = 23.62) showed a significantly higher level of verbal IQ (*T* = 3.49, df = 44, *p* < 0.01) than insecure ones (*M* = 79.67, *SD* = 20.05). Hence, we decided to include verbal IQ as a covariate in the regression model.

#### Adoptive Mothers

The statistical analyses using maternal AAI categories were conducted using a free-autonomous vs. non-free-autonomous partition with dismissing mothers included in the non-free-autonomous group together with unresolved ones. Mothers’ AAI classifications were not associated with age (*p* = 0.87), years of education (*p* = 0.28), length of marriage (*p* = 0.16), or level of psychopathological symptoms (*p* = 0.13).

### Attachment IWMs in Adolescent-Mothers Dyads

#### Classifications

**Table 2** presents the distributions of the attachment classifications of adoptive mothers’ AAI and late-adopted adolescents’ FFI.

**Table 2:** The concordance between adolescents’ FFI and maternal AAI classifications^1^.

	Adoptive mothers’ AAI						
		*F/A*	*Ds*	*P*	*U/D*	Total
Late-adopted adolescents’ FFI	*S*	26	2	0	3	31
	*Ds*	8	2	0	2	12
	*P*	1	0	0	2	3
	*D*	0	0	0	0	0
	Total	35	4	0	7	46

^1^The number of adoptive mothers was counted on the base of the number of their children (*N* = 46; e.g., if a mother had two children, she was counted twice).

We found a significant of 70% (32 dyads) on the two-way AAI and FFI systems (rphi = 0.31, *p* = 0.04) and a concordance of 61% (28 dyads) on the four-way system approaching significance (χ^2^ = 8.29; df = 4, *p* = 0.08). To further examine the possibility of interaction, we conducted multinomial logistic regression analyses predicting adolescents’ secure–insecure FFI categories from mothers’ AAI free-autonomous vs. not-free-autonomous, adding verbal IQ as a covariate. The multinomial logistic regression model indicated that free-autonomous adoptive mothers showed a tendency to have secure late-adopted children (β = 1.42, *p* = 0.08), although adolescents’ high verbal IQ appeared to be a better predictor of their secure classification (β = 0.05, *p* = 0.01).

#### Scales

**Table 3** shows the correlations between the FFI and the AAI state-of-mind scales.

**Table 3:** Spearman’s correlations between the AAI and the FFI scales.

	Adoptive mothers							
	AAI scales of states of mind							
	Late-adopted adolescents	Coherence transcript	Coherence of mind	Idealizing mt	Idealizing ft	Lack of memory	Passivity	Unresolved loss
FFI scales	Coherence-truth	0.10	0.05	-0.20	-0.37^∗∗^	0.03	0.17	-0.16
	Coherence-econ.	0.39^∗∗^	0.33^∗^	-0.13	-0.20	0.15	0.01	-0.24
	Coherence-relation	0.32^∗^	0.27^∗^	-0.16	-0.29^∗^	-0.03	0.08	-0.25^∗^
	Coherence-Manner	0.33^∗^	0.21	-0.20	-0.39^∗∗^	-0.08	-0.14	-0.03
	Overall Coherence	0.30^∗^	0.22	-0.20	-0.37^∗∗^	0.05	0.03	-0.20
	Safe haven Mt	0.23	0.12	-0.14	-0.28^∗^	-0.30^∗^	0.10	-0.00
	Safe haven Ft	0.27^∗^	0.28^∗^	-0.17	-0.37^∗∗^	0.01	0.01	-0.04
	Secure	0.30^∗^	0.29^∗^	-0.17	-0.31^∗^	0.05	0.03	-0.35^∗∗^
	Dismissing	-0.12	-0.12	0.22	0.31^∗^	-0.07	-0.24	0.04
	Preoccupied	-0.11	-0.10	-0.31^∗^	-0.13	0.07	0.23	0.11
	Disorganized	-0.37^∗∗^	-0.32^∗^	0.14	0.28^∗^	0.12	0.11	0.05

^∗^*p* < 0.05, ^∗∗^*p* < 0.01.

The FFI scales were highly correlated with verbal IQ scorings (*r* between 0.35 and 0.63, *p* < 0.01), while no correlations were found between maternal AAI scale of states of mind and adolescents’ verbal IQ scorings (*r* between -0.17 and 0.29, *p* = ns). Based on these correlations, we ran two linear regression analyses (**Table 4**). The first entered the secure pattern as the dependent measure and the second entered the disorganized pattern as the dependent variable. For both regressions, we inserted coherence of transcript, coherence of mind, and idealizing father as independent variables; for the first two, we added unresolved loss in the independent blocks. Verbal IQ was added as a covariate.

**Table 4:** Regression model predicting adolescents’ secure and disorganized FFI scales from maternal AAI states-of-mind scales.

Factor	Standardized β	*t*	*p*
**Secure pattern**			
AAI: coherence of transcript	1.352	2.084	0.044
AAI: coherence of mind	1.286	2.003	0.052
AAI: idealizing ft	-0.145	-1.007	0.320
AAI: unresolved loss	-0.408	-3.271	0.002
Verbal IQ	0.601	5.788	0.000
**Disorganized pattern**			
AAI: coherence of transcript	-0.875	-1.119	0.270
AAI: coherence of mind	-0.611	-0.819	0.418
AAI: idealizing ft	0.011	0.056	0.955
Verbal IQ	-0.307	-2.128	0.039

The regression summary indicated that high coherence of transcript and low unresolved loss of adoptive mothers could predict high secure attachment of their late-adopted children, even if the adolescents’ verbal intelligence made a significant contribution to their prediction. Moreover, high cognitive status of adoptees made a significant contribution to the prediction of low scores on their disorganized patterns of attachment.

## Discussion

Our sample of late-adopted adolescents was classified through the FFI with 67.4% as secure, 26.1% as dismissing, 6.5% as preoccupied, and none disorganized, showing attachment representation distribution overlap with those both from the Italian AAI meta-analysis of non-clinical adolescent samples (Cassibba et al., 2013, 62% free-autonomous, 24% dismissing, 10% preoccupied, and 4% unresolved), and adoption studies using the AICA (Riva Crugnola et al., 2009) and the FFI (Barcons et al, 2012; Stievenart et al., 2012). These data confirms that adoption can be seen as a positive intervention also for late-adopted children who were considered a high risk group due to their adverse pre-adoption experiences.

Concerning the distribution of AAI classifications of adoptive mothers, we found a high percentage of free-autonomous classifications (70%), similar to those in the Italian AAI meta-analysis of non-clinical mothers (Cassibba et al., 2013, 62% free-autonomous, 24% dismissing, 10% preoccupied, and 4% unresolved-disorganized), post-adoption studies including parents assessment (Steele et al., 2008, 2010; Barone and Lionetti, 2012; Pace et al., 2012), and studies on couples seeking to adopt (Cavanna et al., 2011; Calvo et al., 2015; Pace et al., 2015a).

We found a significant concordance between the attachment states of mind of adoptive mothers using the AAI and the attachment representations of late-adopted adolescents using the FFI (70% for two-way and 61% for four-way attachment classification). This result confirmed findings from most of the studies on adoptions, although some research did not find significant associations (**Table 5**).

**Table 5:** Studies assessing attachment concordance between adopted children and their mothers.

Adoption studies	*N* dyads	Children’s age at assessment	Mothers’ attachment measure	Child’s attachment measure	Concordance/correlations
Dozier et al., 2001	50	12–24 months	AAI	Strange situation procedure (SSP)	72% (two ways), *k* = 0.43, *p* < 0.01
Veríssimo and Salvaterra, 2006	106	1–4 years	Secure base scripts	AQS	*r* = 38, *p* < 0.01
Steele et al., 2008	58	4–8 years	AAI	Story stem	*r* = -0.29 (child insec.), *p* < 0.05; *r* = -0.36 (child disorgan.), *p* < 0.01
Riva Crugnola et al., 2009	35	10–15 years	AAI	AICA	63%, (two ways), n.s.
Barone and Lionetti, 2012	20	3–5 years	AAI	MCAST	80%, *p* < 0.01 (two ways)
Pace and Zavattini, 2011	20	4–7 years	AAI	Separation-reunion	60% (two ways), n.s.
Pace et al., 2012	28	4–8 years	AAI	MCAST	56% (two ways), n.s.
Lionetti, 2014	30	1–13 months	AAI	SSP	60% (three ways), *p* = 0.08; 53% (four-way), *p* = 0.01

We would suggest that maternal attachment states of mind, characterized by highly coherent narratives of their own attachment relationships, could be very beneficial for their children via several pathways. First, attachment security of mothers in the AAI is usually associated with both physical and emotional availability, responsiveness, acceptance, and cooperativeness (Allen et al., 2003; Riva Crugnola et al., 2009; Scherf et al., 2013). We would suggest that these maternal behaviors may teach children to feel confident in considering their own and others’ feelings, and to build their own security and self–esteem. Second, free-autonomous adoptive mothers may be more capable of managing and tolerating their children’s separation process during adolescence without experiencing adolescents’ autonomy and exploration behaviors as an attack on the mother–child relationship. Third, free and autonomous adoptive mothers, who are able to coherently integrate their own past attachment history, may be especially good at helping their children to process their early negative and traumatizing experiences and integrate them coherently into their personal biography (Pace et al., 2012). Palacios and Brodzinsky (2010) pointed out that the construction of personal identity becomes even more significant for adopted teenagers, since, during adolescence, connecting the past, the present, and the future in a single and coherent story becomes central and requires the processing and integration of their own story both in adopted children and their parents. On the other hand, adoptive mothers with insecure attachment states of mind, in our study classified as dismissing or unresolved, mostly had insecure children. These data indicated that mothers with low coherence and high unresolved loss would fail to transmit to their late-adopted children the emotional security required for self-confidence and relationally competence (Scharf et al., 2012), and they could be less capable of reducing the impact of their negative past experiences. However, surprisingly three secure adopted adolescents, despite their free-autonomous secondary classification, had unresolved adoptive mothers. From a clinical perspective, it is interesting to mention two points to explain this counter-intuitive data: on one hand, these adoptive mothers were among the few involved in psychotherapeutic treatment in their young adulthood; on the other hand, the three secure adolescents were among the few in our sample who were placed in foster care after the abandonment from biological parents, without experiencing any institutionalization. We would suggest that these protective factors could reduce the impact of the unresolved states of mind of the adoptive mothers on the attachment representation of their adopted children.

Finally, attachment security is overrepresented in late-adopted adolescents with high verbal cognitive scores. This result, in line with findings on early adopted children (van Londen et al., 2007), seems intriguing at different levels. First, our data may indicate a problem with the discriminant validity of the FFI: the more advanced the child’s verbal abilities are, the better she or he is able to describe and talk about attachment relationships, leading to an overrepresentation of attachment security (Atkinson et al., 1999). However, this interpretation does not seem to be confirmed by a study with non-clinical samples (Steele and Steele, 2005). Moreover, controlling for verbal IQ, the relationship between AAI and FFI remained significant and this favored the content and the discriminant validity of the FFI. A second hypothesis could be that, unlike the adult sample, where attachment representations and verbal intelligence were completely distinct domains (Crowell et al., 1996), among adopted adolescents, attachment may be related to cognitive development, as revealed among non-clinical children (O’Connor and McCartney, 2007; Kerns, 2008; West et al., 2013). Lastly, we would suggest that late-adopted adolescent showing high verbal IQ may represent another factor that can help them build their secure attachment representation together with maternal attachment security (adopted children with high verbal IQ may be able to easily learn new habits in adoptive families, etc.). Further studies are needed to investigate whether the correlation between attachment classifications by the FFI and verbal intelligence also holds in normative samples.

## Limitations and Future Developments

This study had several limitations. First, the restrictive eligibility criteria (absence of children with special needs in the sample, low maternal psychopathology, medium-to-high maternal education level, married couples living together) to take part in the study are a limitation for the generalizability of results and they could explain the low rate of insecure attachment in our sample, which was comparable to the rate of the non-adoptive adolescent population. Second, our sample size was quite small and it was not homogeneous (adolescents’ age, age at adoption, children’ country of origin, etc.). Third, fathers’ assessments were lacking in our study. Fourth, the correlational nature of the research design did not allow causal inferences. Lastly, the voluntary participation of adoptive families might have self-selected more sensitive families. Further research is needed to replicate our findings with larger and more uniform samples, using longitudinal research design, and including fathers’ assessment.

## Conclusion

First our results highlighted a concordance of attachment representations among adoptive mother–child dyads during adolescence, endorsing results of some previous studies (e.g., Barone and Lionetti, 2012) on late-adopted children during childhood, and indicating that a relationship with a secure mother may represent a very beneficial experience for the late-adopted adolescents, despite their hard past-experiences. Second, we found a correlation between adolescents’ attachment security and verbal IQ that deserves to be investigated in further studies to assess whether it also holds in normative samples.

## Conflict of Interest Statement

The authors declare that the research was conducted in the absence of any commercial or financial relationships that could be construed as a potential conflict of interest.

## References

[B1] Ainsworth M. S. Blehar M. C. Waters E. Wall S. (1978). *Patterns of Attachment: A Psychological Study of the Strange Situation.* Hillsdale, NJ: Erlbaum.

[B2] Allen J. P. McElhaney K. B. Land D. J. Kuperminc G. P. Moore C. M. O’Beirne-Kelley H. (2003). A secure base in adolescence: markers of attachment security in the mother-adolescent relationship. *Child Dev.* 74 292–307. 10.1111/1467-8624.t01-1-00536 12625451 PMC5332136

[B3] Armsden G. C. Greenberg M. T. (1987). The inventory of parent and peer attachment: individual differences and their relationship to psychological well-being in adolescence. *J. Youth Adolesc.* 16 427–454. 10.1007/BF02202939 24277469

[B4] Atkinson L. Chisholm V. C. Scott B. Goldberg S. Vaughn B. E. Blackwell J. (1999). “Maternal sensitivity, child functional level, and attachment in Down Syndrome,” in *Atypical Attachment in Infancy and Early Childhood among Children at Developmental Risk. Society for Research in Child Development Monograph* Vol. 238 eds Vondra J. I. Barnett D. (New York, NY: Guilford Press) 45–66. 10.1111/1540-5834.00033 10597542

[B5] Bakermans-Kranenburg M. J. van IJzendoorn M. H. (1993). A psychometric study of the adult attachment interview: reliability and discriminant validity. *Dev. Psychol.* 29 870–880. 10.1037/0012-1649.29.5.870

[B6] Bakermans-Kranenburg M. J. van IJzendoorn M. H. (2009). The first 10,000 adult attachment interviews: distributions of adult attachment representations in clinical and non-clinical groups. *Attach. Hum. Dev.* 11 223–263. 10.1080/14616730902814762 19455453

[B7] Barcons N. Abrines N. Brun C. Sartini C. Fumadó V. Marre D. (2012). Social relationships in children from intercountry adoption. *Child. Youth Serv. Rev.* 34 955–961. 10.1016/j.childyouth.2012.01.028

[B8] Barone L. Lionetti F. (2012). Attachment and emotional understanding: a study on late adopted pre schoolers and their parents. *Child Care Health Dev.* 38 690–696. 10.1111/j.13652214.2011.01296.x 21831126

[B9] Beijersbergen M. D. Juffer F. Bakermans-Kranenburg M. J. van IJzendoorn M. H. (2012). Remaining or becoming secure: parental sensitive support predicts attachment continuity from infancy to adolescence in a longitudinal adoption study. *Dev. Psychol.* 48 1277–1282. 10.1037/a0027442 22369333

[B10] Bosmans G. Braet C. van Leeuwen K. Beyers W. (2006). Do parenting behaviors predict externalizing behavior in adolescence, or is attachment the neglected 3rd factor? *J. Youth Adolesc.* 35 354–364. 10.1007/s10964-005-9026-1

[B11] Bowlby J. (1969). *Attachment. Attachment and Loss: Loss*, Vol. 1 New York, NY: Basic Books

[B12] Bretherton I. Munholland K. A. (2008). “Internal working models in attachment relationships: elaborating a central construct in attachment theory,” in *Handbook of Attachment: Theory, Research, and Clinical Applications* 2nd Edn eds Cassidy J. Shaver P. R. (New York, NY: Guilford Press) 102–127.

[B13] Calvo V. Palmieri A. Codamo A. Scampoli M. R. Bianco F. (2015). Perceptions of parental bonding, adult attachment, and marital adjustment in prospective adoptive parents. An empirical study in the pre-adoptive period. *Sex. Relationsh. Ther.* 1–14. 10.1080/14681994.2014.1001355

[B14] Cassibba R. Sette G. Bakermans-Kranenburg M. J. van IJzendoorn M. H. (2013). Attachment the Italian way. *Eur. Psychol.* 18 47–58. 10.1027/1016-9040/a000128

[B15] Cavanna D. Migliorini L. Napoli C. (2011). Attachment and adjustment of pre-adoptive parents. *Int. J. Child Fam. Welf.* 14 148–160.

[B16] Crowell J. A. Waters E. Treboux D. O’Connor E. Colon Downs C. Feider O. (1996). Discriminant validity of the adult attachment interview. *Child Dev.* 67 2584–2599. 10.2307/1131642 9022258

[B17] Dazzi N. Zavattini G. C. (2011). Attachment paradigm and the clinical practice. *G. Ital. Psicol.* 38 729–756. 10.1421/36100

[B18] Derogatis L. R. (1994). *The Symptom Checklist 90-R: Administration, Scoring and Procedures Manual* 3rd Edn Minneapolis, MN: National Computing Systems.

[B19] Dozier M. Rutter M. (2008). “Challenges to the development of attachment relationships faced by young children in foster and adoptive care,” in *Handbook of Attachment: Theory, Research and Clinical Applications* 2nd Edn eds Cassidy J. Shaver P. R. (New York, NY: Guilford Press) 698–717.

[B20] Dozier M. Stovall K. C. Albus K. E. Bates B. (2001). Attachment for infants in foster care: the role of caregiver state of mind. *Child Dev.* 72 1467–1477. 10.1111/1467-8624.00360 11699682

[B21] Escobar M. J. Santelices M. P. (2013). Attachment in adopted adolescents. National adoption in Chile. *Child. Youth Serv. Rev.* 35 488–492. 10.1016/j.childyouth.2012.12.011

[B22] Gallarin M. Alonso-Arbiol I. (2012). Parenting practices, parental attachment and aggressiveness in adolescence: a predictive model. *J. Adolesc.* 35 1601–1610. 10.1016/j.adolescence.2012.07.002 22854650

[B23] Gamble S. A. Roberts J. E. (2005). Adolescents’ perceptions of primary caregivers and cognitive style: the roles of attachment security and gender. *Cogn. Ther. Res.* 29 123–141. 10.1007/s10608-005-3160-7

[B24] Groza V. Muntean A. Ungureanu R. (2012). The adoptive family within the Romanian cultural context: an exploratory study. *Adopt. Q.* 15 1–17. 10.1080/10926755.2012.661327

[B25] Hodges J. Steele M. Hillman S. Henderson K. Kaniuk J. (2003). Changes in attachment representations over the first year of adoptive placement: narratives of maltreated children. *Clin. Child Psychol. Psychiatry* 8 351–367. 10.1177/1359104503008003006

[B26] Hoeve M. Dubas J. S. Gerris J. R. van der Laan P. H. Smeenk W. (2011). Maternal and paternal parenting styles: unique and combined links to adolescent and early adult delinquency. *J. Adolesc.* 34 813–827. 10.1016/j.adolescence.2011.02.004 21397317

[B27] Howe D. (1998). *Patterns of Adoption. Nature, Nurture and Psychosocial Development.* Oxford: Blackwell Science.

[B28] Italian Commission for International Adoptions (2013). *Data and Perspectives in the Adoption International.* Available at: http://www.commissioneadozioni.it/it/per-una-famiglia-adottiva/rapporto-statistico.aspx

[B29] Juffer F. van IJzendoorn M. H. (2005). Behavior problems and mental health referrals of international adoptees: a meta-analytic approach. *J. Am. Med. Assoc.* 293 2501–2515. 10.1001/jama.293.20.2501 15914751

[B30] Kaniuk J. Steele M. Hodges J. (2004). Report on a longitudinal research project, exploring the development of attachments between older, hard-to-place children and their adopters over the first two years of placement. *Adopt. Foster.* 28 61–67. 10.1177/030857590402800208

[B31] Karavasilis L. Doyle A. B. Markiewicz D. (2003). Associations between parenting style and attachment to mother in middle childhood and adolescence. *Int. J. Behav. Dev.* 27 153–164. 10.1080/0165025024400015

[B32] Kerns K. A. (2008). “Attachment in middle childhood,” in *Handbook of Attachment: Theory, Research and Clinical Applications*, 2nd Edn, eds Cassidy J. Shaver P. R. (New York, NY: Guilford Publications) 366–382.

[B33] Kriss A. Steele H. Steele M. (2012). Measuring attachment and reflective functioning in early adolescence: an introduction to the friends and family interview. *Res. Psychother.* 15 87–95. 10.7411/RP.2012.009

[B34] Lionetti F. (2014). What promotes secure attachment in early adoption? The protective roles of infants’ temperament and adoptive parents’ attachment. *Attach. Hum. Dev.* 16 573–589. 10.1080/14616734.2014.959028 25255300

[B35] Main M. Hesse E. Goldwyn R. (2008). “Studying differences in language usage in recounting attachment history: an introduction to the AAI,” in *Clinical Applications of the Adult Attachment Interview* eds Steele H. Steele M. (New York, NY: Guilford Press) 31–68

[B36] O’Connor E. McCartney K. (2007). Attachment and cognitive skills: an investigation of mediating mechanisms. *J. Appl. Dev. Psychol.* 28 458–476. 10.1016/j.appdev.2007.06.007

[B37] Orsini A. Picone L. (2006). *Contributo alla Taratura Italiana di Wechsler Intelligence Scale for Children (WISC-III) [Contribution to the Italian Validation of Wechsler Intelligence Scale for Children].* Firenze: Giunti OS.

[B38] Pace C. S. (2014). Assessing attachment representations among adoptees during middle childhood and adolescence with the Friend and Family Interview (FFI): clinical and research perspectives. *Front. Psychol.* 5:1114 10.3389/fpsyg.2014.01114 PMC4181240 25324815

[B39] Pace C. S. San Martini P. Zavattini G. C. (2011). The factor structure of the Inventory of Parent and Peer Attachment (IPPA): a survey of Italian adolescents. *Pers. Individ. Differ.* 51 83–88. 10.1016/j.paid.2011.03.006

[B40] Pace C. S. Santona A. Zavattini G. C. Di Folco S. (2015a). Attachment states of mind and couple relationships in couples seeking to adopt. *J. Child Fam. Stud.* 1–13. 10.1007/s10826-015-0134-6

[B41] Pace C. S. Zavattini G. C. Tambelli R. (2015b). Does Family Drawing assess attachment representations of late-adopted children? A preliminary report. *Child Adolesc. Ment. Health* 20 26–33. 10.1111/camh.12042 32680327

[B42] Pace C. S. Zavattini G. C. (2011). ‘Adoption and attachment theory’ the attachment models of adoptive mothers and the revision of attachment patterns of their late-adopted children. *Child Care Health Dev.* 37 82–88. 10.1111/j.13652214.2010.01135.x 20637017

[B43] Pace C. S. Zavattini G. C. D’Alessio M. (2012). Continuity and discontinuity of attachment patterns: a short-term longitudinal pilot study using a sample of late-adopted children and their adoptive mothers. *Attach. Hum. Dev.* 14 45–61. 10.1080/14616734.2012.636658 22191606

[B44] Palacios J. Brodzinsky D. (2010). Adoption research: trends, topics, outcomes. *Int. J. Behav. Dev.* 34 270–284. 10.1177/0165025410362837

[B45] Riva Crugnola C. Sagliaschi S. Rancati I. (2009). Attachment quality and elaboration of early unfavourable experiences in adopted preteens. *Psicol. Clin. Sviluppo* 13 515–542. 10.1449/30785

[B46] Rosenstein D. S. Horowitz H. A. (1996). Adolescent attachment and psychopathology. *J. Consult. Clin. Psychol.* 64 244 10.1037/0022-006X.64.2.244 8871408

[B47] Rutter M. O’Connor T. G. (2004). Are there biological programming effects for psychological development? Findings from a study of Romanian adoptees. *Dev. Psychol.* 40 81–94. 10.1037/0012-1649.40.1.81 14700466

[B48] Scharf M. Mayseless O. Kivenson-Baron I. (2012). Intergenerational concordance in Adult Attachment Interviews with mothers, fathers and adolescent sons and subsequent adjustment of sons to military service. *Attach. Hum. Dev.* 14 367–390. 10.1080/14616734.2012.691652 22697470

[B49] Scherf K. S. Smyth J. M. Delgado M. R. (2013). The amygdala: an agent of change in adolescent neural networks. *Horm. Behav.* 64 298–313. 10.1016/j.yhbeh.2013.05.011 23756154 PMC3781589

[B50] Siegel S. Castellan N. J. Jr. (1988). *Non Parametric Statistics for the Behavioral Science.* New York, NY: McGraw-Hill Book Company.

[B51] Steele H. Steele M. (2005). “The construct of coherence as an indicator of attachment security in middle childhood: the friends and family interview,” in *Attachment in Middle Childhood*, eds Kerns K. A. Richardson R. A. (New York, NY: Guilford Press) 137–160.

[B52] Steele H. Steele M. Fonagy P. (1996). Associations among attachment classifications of mothers, fathers, and their infants. *Child Dev.* 67 541–555. 10.2307/1131831 8625727

[B53] Steele M. Henderson K. Hodges J. Kaniuk J. Hillman S. Steele H. (2007). “In the best interests of the late-placed child: a report from the attachment representations and adoption outcome study,” in *Developmental Science and Psychoanalysis: Integration and Innovation*, eds Mayes L. C. Fonagy P. Target M. (London: Karnac Books) 159–182.

[B54] Steele M. Hodges J. Kaniuk J. Hillman S. Henderson K. (2003). Attachment representations and adoption: associations between maternal states of mind and emotion narratives in previously maltreated children. *J. Child Psychother.* 29 187–205. 10.1080/0075417031000138442

[B55] Steele M. Hodges J. Kaniuk J. Steele H. (2010). Mental representation and change: developing attachment relationships in an adoption context. *Psychoanal. Inq.* 30 25–40. 10.1080/07351690903200135

[B56] Steele M. Hodges J. Kaniuk J. Steele H. Hillman S. Asquith K. (2008). “Forecasting outcomes in previously maltreated children. The use of the AAI in a longitudinal adoption study,” in *Clinical Applications of the Adult Attachment Interview* eds Steele H. Steele M. (New York, NY: Guilford Press) 427–451.

[B57] Stievenart M. Casonato M. Muntean A. van de Schoot R. (2012). The friends and family interview: measurement invariance across Belgium and Romania. *Eur. J. Dev. Psychol.* 9 737–743. 10.1080/17405629.2012.689822

[B58] Stievenart M. Roskam I. Meunier J. C. Van de Moortele G. (2011). The reciprocal relation between children’s attachment representations and their cognitive ability. *Int. J. Behav. Dev.* 35 58–66. 10.1177/0165025410370790

[B59] Thompson R. A. (1999). “Early attachment and later development,” in *Handbook of Attachment: Theory, Research, and Clinical Applications* eds Cassidy J. Shaver P. R. (New York, NY: Guilford Press) 265–286.

[B60] Tini M. Corcoran D. Rodrigues-Doolabh L. Waters E. (2003). Maternal attachment scripts and infant security. *Paper Presented at the Biennial Meeting of the Society for Research in Child Development*, Tampa, FL.

[B61] van den Dries L. Juffer F. van IJzendoorn M. H. Bakermans-Kranenburg M. J. (2009). Fostering security? A meta-analysis of attachment in adopted children. *Child. Youth Serv. Rev.* 31 410–421. 10.1016/j.childyouth.2008.09.008

[B62] van IJzendoorn M. (1995) Adult attachment representations, parental responsiveness, and infant attachment: a meta-analysis on the predictive validity of the Adult Attachment Interview. *Psychol. Bull*. 117 387–403. 10.1037/0033-2909.117.3.387 7777645

[B63] van IJzendoorn M. H. Juffer F. (2006). The Emanuel Miller Memorial Lecture 2006: adoption as intervention. Meta-analytic evidence for massive catch-up and plasticity in physical, socio-emotional, and cognitive development. *J. Child Psychol. Psychiatry* 47 1228–1245. 10.1111/j.1469-7610.2006.01675.x 17176378

[B64] van IJzendoorn M. H. Schuengel C. Bakermans-Kranenburg M. J. (1999). Disorganized attachment in early childhood: meta-analysis of precursors, concomitants, and sequelae. *Dev. Psychopathol.* 11 225–249. 10.1017/S0954579499002035 16506532

[B65] van Londen W. M. Juffer F. van IJzendoorn M. H. (2007). Attachment, cognitive, and motor development in adopted children: short-term outcomes after international adoption. *J. Pediatr. Psychol.* 32 1249–1258. 10.1093/jpepsy/jsm062 17709336

[B66] Velotti P. Castellano R. Zavattini G. C. (2011). Adjustment of couples following childbirth: the role of generalized and specific states of mind in an italian sample. *Eur. Psychol.* 16 1–10. 10.1027/1016-9040/a000022

[B67] Verhage M. L. Schuengel C. Madigan S. Fearon R. M. P. Oosterman M. Cassibba R. (2015). “Intergenerational transmission of attachment and mediation by caregiver sensitivity: new meta-analytic findings,” in *Paper Presented at the Biennial Meeting of the Society for Research in Child Development*, Philadelphia, PA.

[B68] Veríssimo M. Salvaterra F. (2006). Maternal secure-base scripts and children’s attachment security in an adopted sample. *Attach. Hum. Dev.* 8 261–273. 10.1080/14616730600856149 16938707

[B69] Waters E. (1995). Appendix a: the attachment q-set (version 3.0*)*. *Monogr. Soc. Res. Child Dev.* 60 234–246. 10.1111/j.1540-5834.1995.tb00214.x

[B70] Waters H. S. Waters E. (2006). The attachment working models concept: among other things, we build script-like representations of secure base experiences. *Attach. Hum. Dev.* 8 185–197. 10.1080/14616730600856016 16938702

[B71] Wechsler D. (1991). *The Wechsler Intelligence Scale for Children* 3rd Edn San Antonio, TX: The Psychological Corporation

[B72] West K. K. Mathews B. L. Kerns K. A. (2013). Mother–child attachment and cognitive performance in middle childhood: an examination of mediating mechanisms. *Early Child. Res. Q.* 28 259–270. 10.1016/j.ecresq.2012.07.005

[B73] West M. Adam K. Spreng S. Rose S. (2001). Attachment disorganization and dissociative symptoms in clinically treated adolescents. *Can. J. Psychiatry* 46 627–631. 11582824 10.1177/070674370104600707

[B74] Zimmerman P. Fremmer-Bombik E. Spangler G. Grossman K. E. (1997). “Attachment in adolescence: a longitudinal perspective,” in *Development of Interaction and Attachment: Traditional and Non-Traditional Approaches* eds Koops W. Hoeksema J. B. van den Boom D. C. (Amsterdam: North-Holland) 281–291.

